# Editorial: Rhinitis and pollution

**DOI:** 10.3389/falgy.2025.1735318

**Published:** 2025-11-13

**Authors:** Herberto J. Chong-Neto, R. Maximiliano Gómez

**Affiliations:** 1 Federal University of Paraná, Curitiba, Brazil; 2 Catholic University of Salta, Salta, Argentina

**Keywords:** allergic rhinitis, asthma, exposome, pollution, sick building syndrome

In this volume you will find the description of a topic that must warn the planetary population about the environment we have (a consequence of what we do), and the impact in human health. Here, we focus in the allergy field.

*Revisiting the Air–Allergy Interface: From Pollutant Adjuvancy to Planetary Health*, the compilation of the four recent articles published in Frontiers in Allergy—Rosario et al., González-Díaz et al., Zhong et al., and Shusterman—are collectively mapping an evolving frontier in allergy research. Together, they show how pollution acts not only as an irritant or trigger, but as a true biological amplifier of sensitization—a concept with both historical and modern mechanistic foundations.

Shusterman reconstructs the intellectual lineage of the pollutant-adjuvant concept “*From Allergy versus Irritation to Adjuvancy: Lessons from History”*. Beginning with Japanese and Californian studies from the 1980s, he recounts how diesel exhaust particles enhanced allergen-specific IgE responses and even induced sensitization to new, otherwise innocuous antigens. Subsequent decades broadened this paradigm: second-hand smoke, wood-smoke, formaldehyde, phthalates, and ozone–limonene reaction products all demonstrated adjuvant-like effects.

In “*The Modern Scene: Pollution and Rhinitis as Markers of the Anthropocene”*, Rosario et al. portray rhinitis as a sentinel disease of the Anthropocene, where nasal epithelium functions as a biosensor against particulate matter, ozone, and nitrogen oxides, activating alarmins (IL-25, IL-33, TSLP) and amplifying type 2 inflammation. Rising CO₂ and warming extend pollen seasons and allergenicity, creating dual chemical and biological adjuvants acting on airway barriers.

Zhong et al. demonstrate “*Causality and Genetics: The Mendelian Lens”*, through Mendelian randomization using UK Biobank and FinnGen data, a causal relationship between PM₁₀ exposure and asthma, though not with allergic rhinitis, implying distinct biological pathways despite overlapping epidemiology.

Also, González-Díaz et al. analyzed 402 urban residents and found that 91% reported at least one sick-building-syndrome symptom in “*Indoor–Outdoor Continuum: Sick Building Syndrome and Hidden Pollutants”*. These correlated with outdoor PM₂.₅, nitrogen oxides, and airborne pollen, showing that indoor air mirrors outdoor pollution and forms an indoor adjuvancy environment.

“*Synthesizing Across Scales: From Molecules to Buildings”* we find four studies, altogether defining an eco-immunologic continuum linking pollutant adjuvants, climate drivers, and host genetics. Airway allergy becomes a mirror of environmental change, where epidemiology, immunology, and architecture intersect.

The “*Future Horizons: Toward Adjuvant-Aware Allergy Prevention”* exhorts us to recognize and act in consequence about priorities, including: (i) unifying oxidative-stress and cytokine models of pollutant adjuvancy (Shusterman); (ii) genotype–exposome mapping (Zhong et al.); (iii) building-level interventions (González-Díaz et al.); (iv) antioxidant and dietary strategies (Shusterman); and (v) integrating allergy prevention with climate mitigation (Rosario et al.).

In conclusion, from diesel exhaust to indoor formaldehyde, from genomic susceptibility to climate-driven pollen shifts, these studies argue that pollution is an active participant in allergic diseases. The airway epithelium translates chemical and biological stressors into inflammation and sensitization. Understanding and mitigating these interactions will define the next decade of respiratory-allergy prevention.

